# Life stage shifts in salivary microbiota determinants: a cross-sectional study in twins

**DOI:** 10.1038/s41522-026-01134-0

**Published:** 2026-09-16

**Authors:** Simon Haworth, Eftychia Bellou, Ralf Kuja-Halkola, Wai-Lok Yau, Ingegerd Johansson, Anders Esberg

**Affiliations:** 1 Department of Odontology, University of Umeå, Umeå, Sweden; 2 Bristol Dental School, University of Bristol, Bristol, UK; 3 Department of Medical Epidemiology and Biostatistics, Karolinska Institutet, Stockholm, Sweden

**Keywords:** Genetics, Microbiology

## Abstract

Both host genetics and environmental exposures shape the salivary microbiota; however, the relative contributions of these factors from early life to adulthood remain unclear. To address this, we profiled the salivary microbiota of 3387 twin pairs: 939 infant pairs (9–12 months) and 2,448 adult pairs (23–52 years) using full-length 16S rRNA gene sequencing. In infants, the shared environment dominated: richness, diversity, and overall community composition were driven primarily by shared environmental effects, accounting for >50% of variance across key metrics. Host genetics had no detectable effect on community composition or species presence and only modest effects on species abundances and predicted functions. By contrast, in adults, the pattern reversed: shared environment contributed little, while host genetics exerted a broader, stronger influence, explaining 19–45% of variance in core diversity metrics and significantly affecting both species’ presence, abundance, and predicted functions. Several clinically relevant oral taxa showed substantial heritability in adults, with roughly one-third of their variance attributable to genetic factors. These results reveal a life-stage shift in determinants of the salivary microbiota and their predicted functions: infancy is largely shaped by environmental exposures, whereas adult communities increasingly reflect host genetic architecture. These findings suggest that interventions to promote healthy oral microbiota may need to be tailored to life-stage.

## Introduction

The oral cavity represents one of the most complex ecosystems where microbial and host factors jointly shape structural and functional traits. Studies on oral microorganisms have identified critical host-microbe interactions in both health and disease. These interactions encompass interplay with conditions inside the oral cavity, such as dental caries and periodontitis. They also affect systems beyond the mouth, including inflammatory and metabolic gut disorders, neurodegenerative diseases, cardiovascular diseases, and rheumatic heart disease (see reviews^1–3^).

From birth, the oral cavity is exposed to a continual influx of microbial taxa. The initial establishment of the indigenous oral microbiota occurs within the first weeks of life, with *Streptococcus* species rapidly becoming dominant^4,5^. As the infant grows, the oral microbiota diversifies and matures through the acquisition of commensal species typically found in adults^6,7^. Maternal transmission and mode of feeding play a central role in this process, with multiple studies demonstrating greater similarity in oral microbiota profiles among mother-child pairs than among unrelated individuals^5,8–10^. Over time, the oral microbiota matures into a diverse ecosystem shaped by host genetics as well as environmental exposures, including diet, lifestyle, oral hygiene, and dental or systemic disease status^11–13^. Yet the relative contributions of genetic and environmental influences across the life course remain incompletely understood.

Twin studies offer a powerful framework for disentangling genetic and environmental influences on microbiota composition. Under the classical twin model, a genetic contribution is inferred when monozygotic (MZ) twins show greater similarity than dizygotic (DZ) twins, assuming comparable shared environments. With sufficiently large cohorts, the variance attributable to additive genetic and environmental components can be estimated^14^. Previous research on the heritability of the oral microbiota has yielded inconsistent findings, with some emphasizing strong environmental effects^8,15–18^, and others demonstrating substantial heritability^19–22^. The largest study to date (752 twin pairs aged 11–24 years) reports heritability estimates exceeding 50% for several salivary microbiota traits, surpassing those reported for the gut microbiota^19,23^. Consequently, one primary need is to evaluate the proportion attributed to environmental factors and host genetics in the complex oral microbiota in a larger setting than studied so far, and evaluate if the proportions change with age.

To address this knowledge gap, we leverage a large, well-characterized salivary microbiota twin cohort, comprising 3387 twin pairs, including 939 twin pairs from infants and 2448 adult twin pairs. This design enables estimation of both heritability and environmental contributions within each cohort separately and allows us to contrast age-associated patterns between early life and adulthood.

## Results

### Characteristics of the NewTwin and the STAGE cohorts

**Fig. 1:**
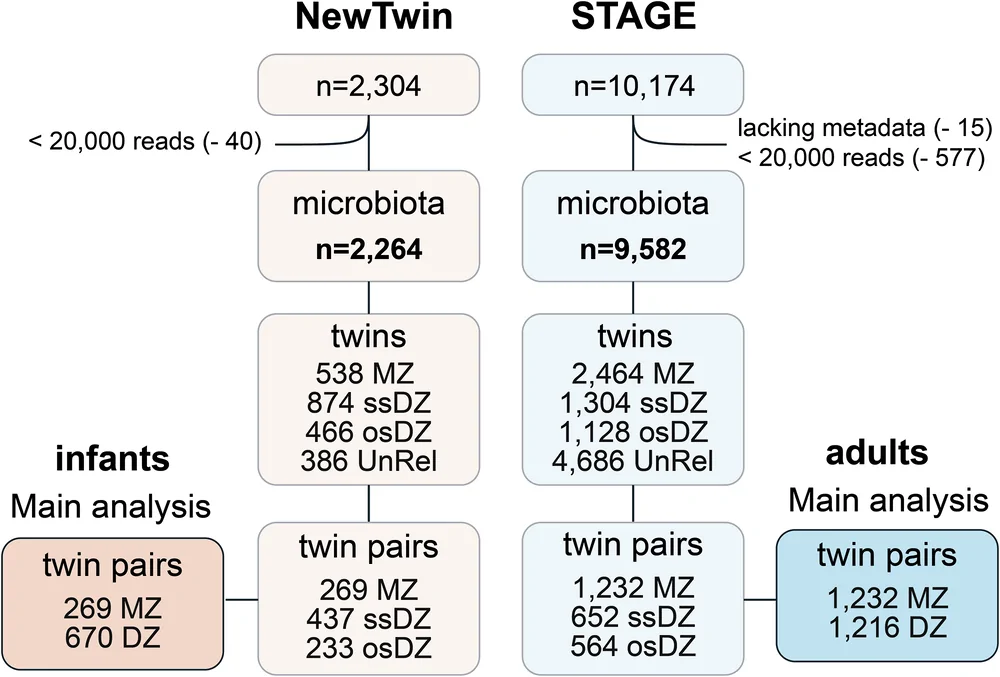
Study flow chart. The chart illustrates the total number of participants with saliva-derived DNA available for microbiota sequencing in the Swedish Twin Register “NewTwin” and “STAGE” cohorts, and the numbers remaining after exclusion of individuals with incomplete metadata or fewer than 20,000 sequencing reads, stratified by zygosity. The “NewTwin” participants are referred to as infants in the text and the “STAGE” participants as adults. MZ = monozygotic twins; DZ = dizygotic twins; ss = same-sex twin pairs; os = opposite-sex twin pairs; UnRel = unrelated twins (excluded from further use within this study based on the requirements of full-twin pairs).

The NewTwin group comprised 2304 infants aged 9 to 12 months, of whom 48.3% were girls. Of these, 2264 provided saliva samples, had sufficient DNA, and generated over 20,000 reads for microbiota characterization and were included. The included 939 infant twin pairs were distributed across 269 MZ twin pairs, 437 same sex DZ twin pairs, and 233 opposite sex DZ twin pairs for heritability analyses (Fig. 1).

The STAGE cohort consisted of 10,174 adult twins with an average (min, max) age of 38 years (23, 52), and 60.1% were women. Of these, zygosity determination was missing for 15 participants, and 577 did not reach ≥ 20,000 reads and were excluded. Following quality control, 9582 participants were retained and were distributed into 1232 MZ, 652 same sex DZ, and 564 opposite sex DZ twin pairs for twin heritability analyses (Fig. 1). Characteristics of the two twin cohorts are presented in Supplementary Table 1.

### Microbiota sequencing and overall characteristics

**Fig. 2:**
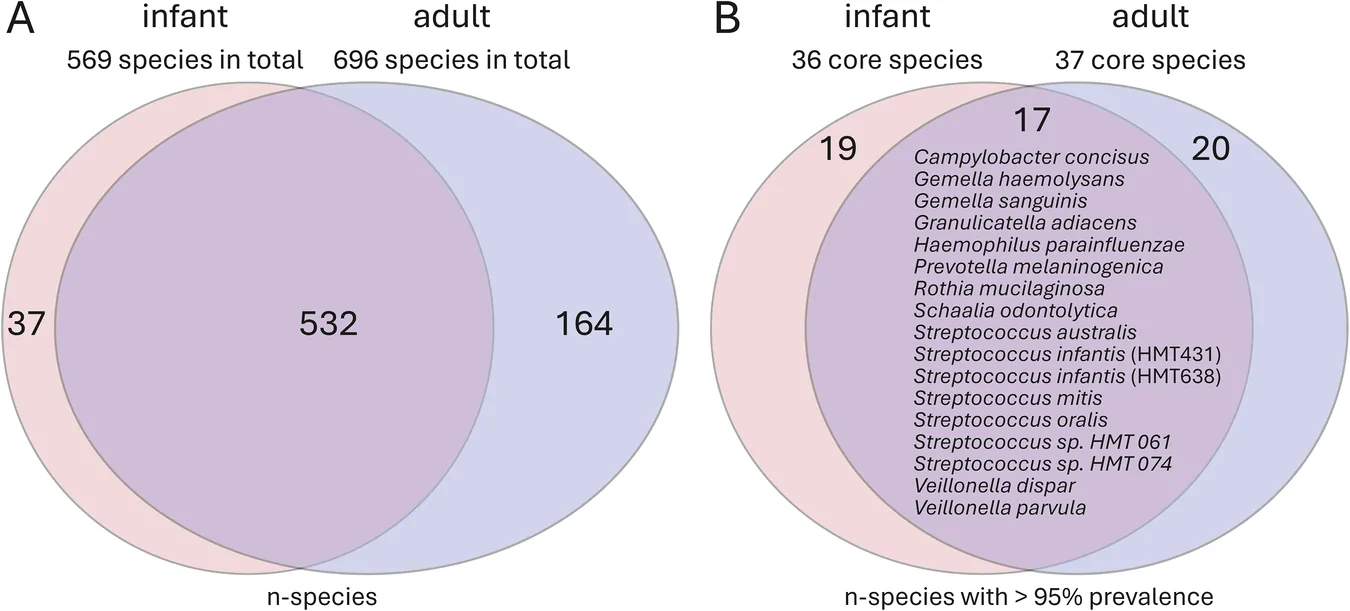
Comparison of total and core salivary microbiota in infants and adults. **A** Venn diagram showing the total number of species identified across both cohorts. In total, 733 species were detected, with 569 species observed in infants and 696 in adults, of which 532 were shared between the cohorts. **B** Venn diagram illustrates the core salivary microbiota, defined as species with a prevalence > 95%. A total of 36 core species were identified in infants and 37 in adults, of which 17 species were shared between the groups.

Full-length 16S rRNA gene sequencing generated a median of 109,506 quality-filtered reads (range [min-max]: 21,984–258,269) in the 2264 infants and 40,851 reads (range 20,005–270,446) in the 9582 adult samples. In total, 733 species/phylotypes were identified across both cohorts. The infants harboured 569 species/phylotypes and adults 696, with 532 shared between the groups (Fig. 2A). After rarefaction to 20,000 reads per sample, infants had a median of 109 observed species per individual (richness), whereas adults had 150 species (*p* < 0.0001, Supplementary Fig. 1A); adults also showed higher Shannon diversity index values (*p* < 0.001, Supplementary Fig. 1B) and the cohorts showed a clear compositional separation (Supplementary Fig. 1C). For clarity, species and phylotypes are collectively referred to as species throughout the text.

A core salivary microbiota of 17 species was shared by infants and adults, each with a prevalence exceeding 95%. This core included *Streptococcus australis*, *Granulicatella adiacens*, *Streptococcus mitis*, *Rothia mucilaginosa*, *Prevotella melaninogenica*, *Veillonella parvula*, *Veillonella dispar*, *Streptococcus infantis (HMT431 and HMT 638)*, *Haemophilus parainfluenzae*, *Streptococcus* sp. HMT 061, *Streptococcus oralis*, *Campylobacter concisus*, *Schaalia odontolytica*, *Streptococcus* sp. HMT 074, *Gemella haemolysans*, and *Gemella sanguinis* (Fig. 2) (Supplementary Table 2). Thus, a stable core oral microbiota appeared established at 9–12 months of age and persisted for at least five decades (Fig. 2B).

Top-ranked taxa more prevalent in infants than adults included *Micrococcus luteus*, *Microbacterium flavescens*, *Novosphingobium panipatense*, *Bosea vestrisii*, *Staphylococcus hominis*, *Dermabacter hominis*, *Moraxella lincolnii*, *Dolosigranulum pigrum*, *Cloacibacterium normanense*, *Lachnospiraceae* [G-2] bacterium, *Acinetobacter lwoffii*, and *Kocuria atrinae*. Many were skin or environmental genera (e.g., *Micrococcus*, *Kocuria*, *Acinetobacter*, *Bosea*, *Cloacibacterium*), whereas others typify the infant nasopharynx or early gut (e.g., *Moraxella*, *Dolosigranulum*, and *Lachnospiraceae* sp.). In contrast, adults were more frequently enriched for species associated with caries and periodontitis, including *Streptococcus mutans, Tannerella forsythia, Treponema denticola, Prevotella denticola, Filifactor alocis*, and *Porphyromonas endodontalis* (Supplementary Table 2). Infant profiles are consistent with early-life, exposure-driven colonization, and niche immaturity, whereas adult profiles reflect established dental plaque ecosystems with greater representation of cariogenic and periodontal taxa. This age gradient aligns with the ecological maturation of the oral cavity.

### Life-stage shift in determinants of species prevalence

We assessed species prevalence and within-pair similarity using both shared-species counts and prevalence-based twin correlations, followed by variance decomposition into additive genetic (A), shared environmental (C), and unique environmental (E) components. To ensure stable estimates, analyses were restricted to species present in 5–95% of individuals (178 species in infants and 289 species in adults). Multiple testing was controlled using Benjamini–Hochberg FDR-adjusted *p*-values.

**Fig. 3:**
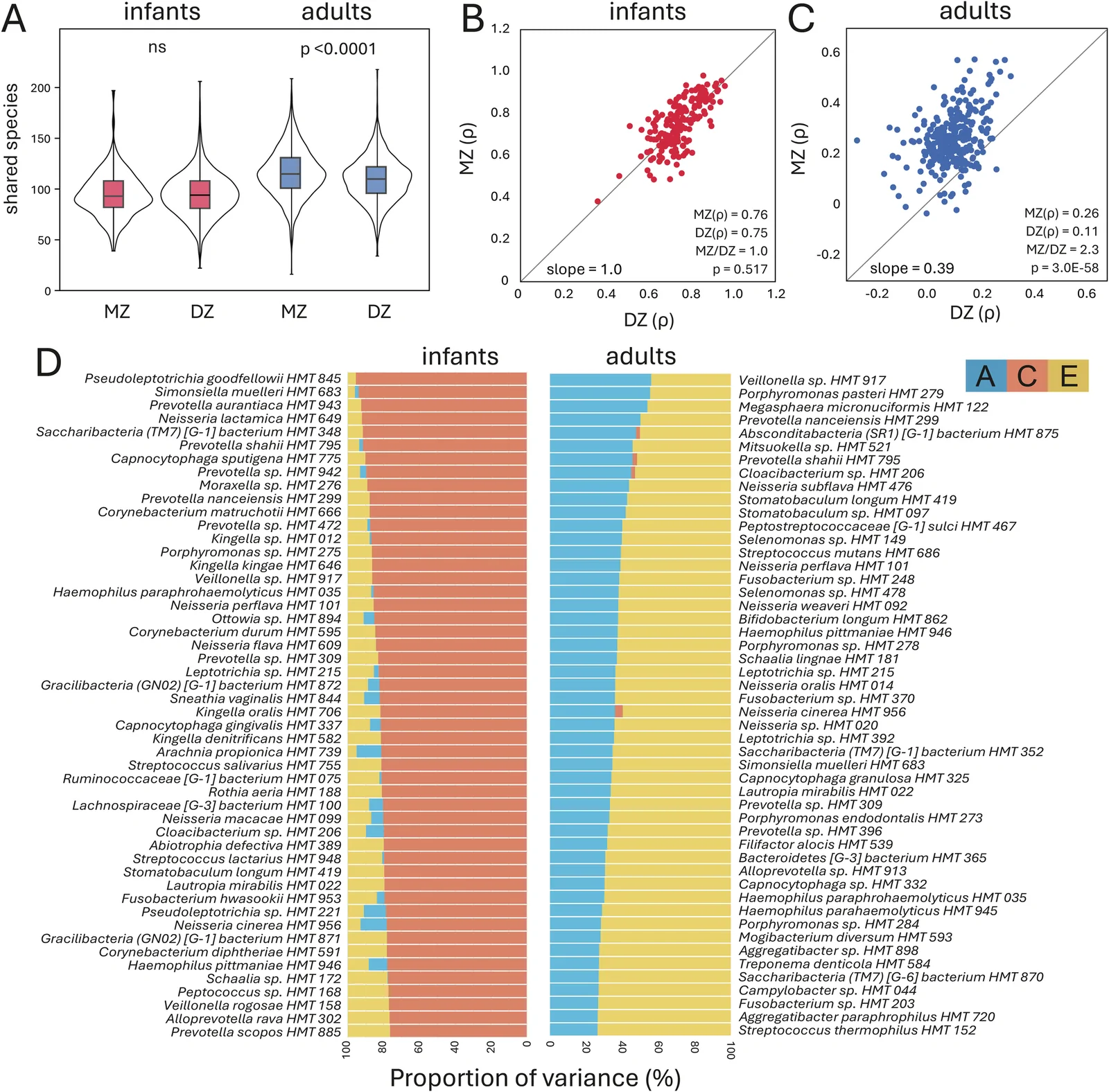
Determinants for microbial species presence in infants and adults. Violin plot with overlaid boxplots showing the number of species shared within-twin-pairs stratified by zygosity (**A**) and 2D scatterplots showing tetrachoric correlations (ρ) for species presence across twin pairs (**B**, **C**). **D** Stacked column plots displaying the 50 species most influenced by shared environment [C] for infants and the 50 most heritable [A] in adults. All species shown were significant after FDR correction. Partition of variance was estimated using liability-threshold ACE models adjusted for sex, sequencing depth, and age in adults. Analyses were restricted to species with prevalence between 5% and 95%, and multiple testing was controlled using the Benjamini–Hochberg procedure. MZ monozygotic twins, DZ dizygotic twins.

In infants, the median number of shared species did not differ by zygosity (*p* > 0.29), with twin pairs sharing approximately 95 species. In contrast, MZ adult twins shared a median of 7 more species than DZ pairs (116 vs. 109, respectively; *p* < 0.0001; Fig. 3A). Tetrachoric correlation coefficients in infants were highly similar between MZ and DZ twin pairs (0.76 vs 0.75, 1.0-fold, *p* = 0.517; Fig. 3B). In adults, tetrachoric correlations revealed, overall lower but higher within-twin-pair similarity among MZ (ρ = 0.26) than DZ twins (ρ = 0.11) for 264 (91%) species (Fig. 3C), with an average difference between MZ and DZ twin-pairs of 2.3-fold (*p* = 3.0E−58).

Liability-threshold ACE models, for species presence, identified shared environmental factors as the primary source of variation in infants, significantly (FDR-*P* < 0.05) explaining prevalence variation for 175 of 178 species (98.3%), with variance components ranging from 41% to 96%. No species showed a significant genetic contribution in their presence (Supplementary Table 3). The 50 species most strongly influenced by shared environmental factors are shown in Fig. 2D. In contrast, liability-threshold ACE models in adults revealed significant additive genetic effects for 62 species (20.8%), accounting for 20% to 56% of the variance in their prevalence (the top 50 species most strongly influenced by additive genetic factors are shown in Fig. 3D), whereas shared environmental influences were minimal (Supplementary Table 4).

All ACE models were adjusted for sex and total number of reads (and age in adult analyses).

### Heritable vs environmental drivers of species abundance across life-stages

We next assessed genetic and environmental influences on species abundance by first quantifying within-pair similarity with species-level intraclass correlations (ICC), followed by decomposing variance into additive genetic (A), shared environmental (C), and unique environmental/measurement error (E) components using univariate twin ACE models. ICCs and ACE models were fit on Yeo-Johnson-transformed species abundances adjusted for sex, sequencing depth, batch (and age additionally included in adult analyses). For ACE fitting, taxa with fewer than 10 complete MZ or DZ pairs were excluded. ACE estimates are interpreted under standard twin-model assumptions (equal environments for MZ and DZ, no substantial assortative mating, and no unmodeled genetic-environmental interactions. Abundance analyses were restricted to taxa present in > 5% of samples (214 species in infants; 324 species in adults). Multiple testing was controlled by Benjamini–Hochberg FDR, and results are reported at FDR < 0.05.

**Fig. 4:**
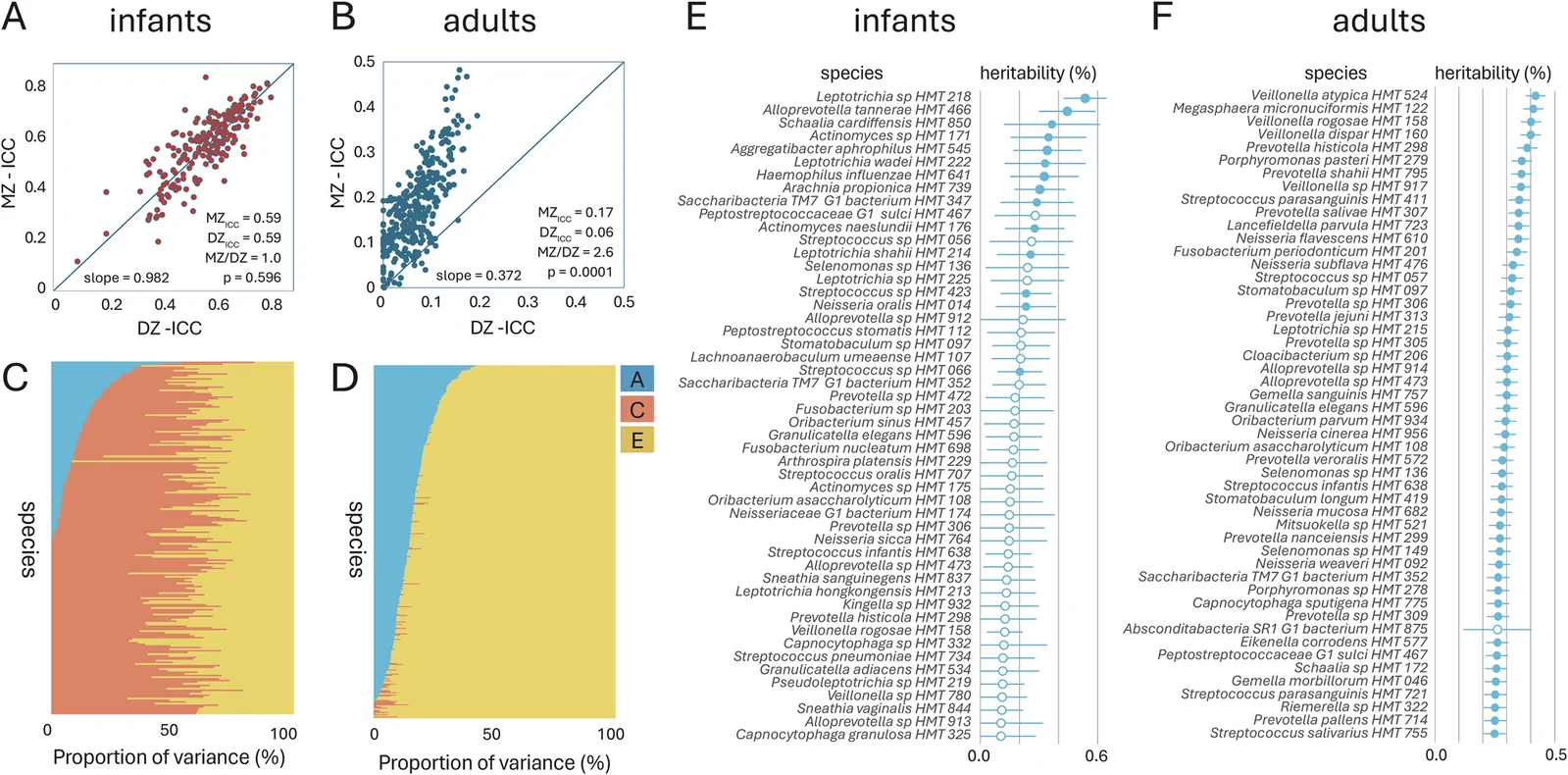
Determinants of species abundance in infants and adults. Species present in > 5% of participants were analysed using intra-class correlation (ICC) in infants (**A**) and adults (**B**). Scatterplots show ICC estimates for monozygotic (MZ) and dizygotic (DZ) twin pairs; *p*-values indicate differences between groups (Mann–Whitney U test). Variance in species abundance was partitioned using ACE models into additive genetic [A], shared environmental [C], and non-shared environmental [E] components for 214 infant species (**C**) and 324 adult species (**D**). The top 50 species with the highest additive genetic contribution [A] are shown for infants in (**E**) and for adults in (**F**). Points represent mean estimates with 95% confidence intervals; filled points indicate significant A components (FDR-adjusted). All models were adjusted for sex, sequencing depth, batch, and additionally for age in adults.

Infants exhibited generally higher ICCs for species abundances than adults (Fig. 4A vs. Fig. 4B, Supplementary Tables 5 and 6), but no difference was observed between MZ and DZ infant twin pairs (MZ = 0.59; DZ = 0.59) (Fig. 4A). Among adults, ICCs were higher in MZ than DZ pairs (MZ = 0.17; DZ = 0.06; *p* = 0.0001), showing greater within-pair similarity in MZ twins (Fig. 4B).

In infants, ACE modelling showed that shared environmental factors significantly influenced the abundance of all but three species (98.6%), accounting for 17% to 69% of their variance (Fig. 4C) (Supplementary Table 5). The ACE models also indicated that heritability significantly contributed to variation in the abundance of 14 species (6.5%), with estimates ranging from 20% to 54%. The 50 species most strongly influenced by additive genetics in infants are shown in Fig. 4E. The five species with the highest heritability (A; %, 95% CI) were *Leptotrichia* sp. *HMT218* (54% [43–64%]), *Alloprevotella tannerae* (44% [30–59%]), *Schaalia cardiffensis HMT 850* (37% [12–61%])*, Actinomyces* sp. *HMT 171* (35% [16-54%]), and *Aggregatibacter aphrophilus* (34% [17-51%]).

Adults showed the opposite pattern; here, heritability dominated over shared environmental factors and significantly influenced species abundance for 259 of the 324 species evaluated (79.9%), with estimates ranging from 5% to 42% (Fig. 4D). The 50 species with the strongest genetic influence are shown in Fig. 4F, with taxa from the genera *Veillonella*, *Prevotella*, *Fusobacterium*, and *Streptococcus* frequently ranking highest. Shared environmental effects were limited in adults, with significant influence observed for five species (C; %, 95% CI): *Actinomyces naeslundii* (1% [0-4%]), *Actinomyces* sp. *HMT170* (2% [0–6%]), *Capnocytophaga* sp. *HMT336* (6% [2–10%]), *Weeksellaceae G1* sp. *HMT931* (6% [2–9%]), and *Aggregatibacter* sp. *HMT512* (15% [11–18%]) (Supplementary Table 6).

### Age-dependent genetic control of salivary microbiota composition

Next, genetic and environmental influences on overall microbiota composition were examined by within-pair dissimilarity based on Aitchison distance for twin pairs and subsequently partitioned and quantified variance components 1 to 3 using twin ACE modelling.

**Fig. 5:**
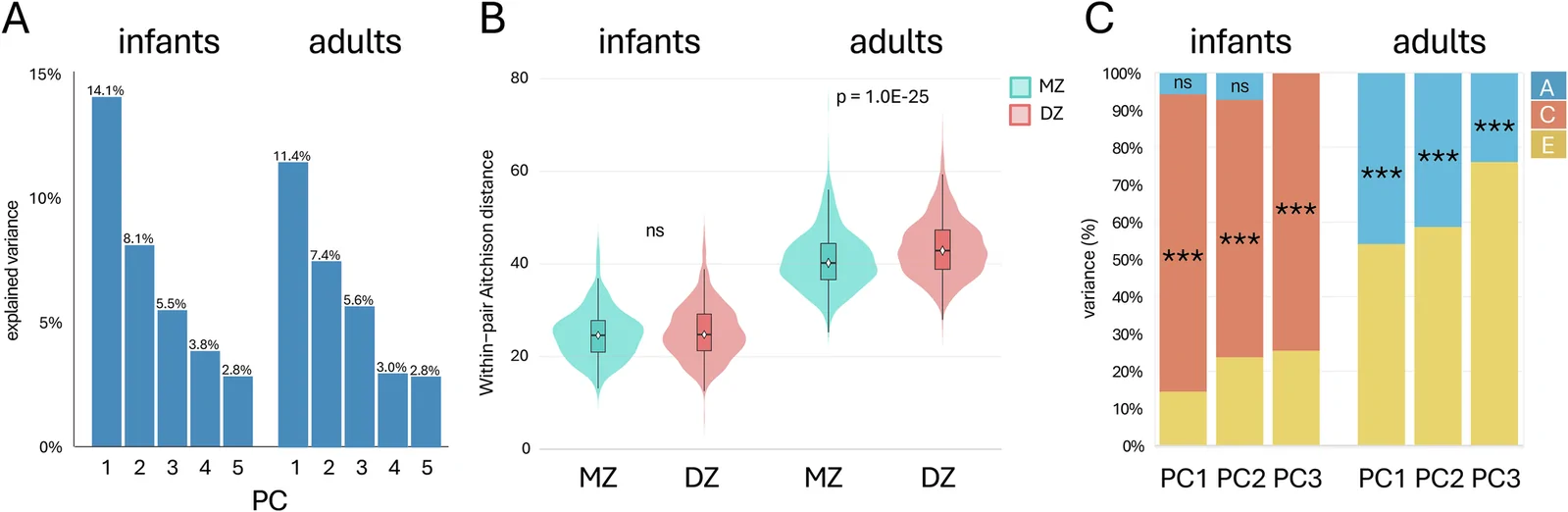
Determinants of microbiota composition within infant and adult twins. Aitchison distance was computed as the Euclidean distance in the adjusted CLR space and the variance explained within the first five principal components (PC) (**A**), within-twin-pair distance (**B**), and PC1-3 partition of variance into additive genetic [A], shared [C], and non-shared [E] environmental determinants using the classical twin modelling (ACE) (**C**). MZ = monozygotic twins; DZ = dizygotic twins. *** = *p* < 0.0001. ns = non-significant.

PCA of Aitchison distances (PC1–20) explained 47% of the compositional variation in infants and 53% in adults (Fig. 5A, showing PC1-5). Infant twin pairs displayed markedly higher microbiota similarity than adult pairs, reflected by shorter within-pair distances (*p* < 0.0001; Fig. 5B). Within infants, Aitchison distances were comparable between MZ and DZ twin pairs (*p* > 0.05), whereas in adults, MZ pairs showed shorter distances than DZ pairs (*p* < 0.0001), consistent with an age-dependent genetic contribution to overall microbiota composition.

To quantify the relative contributions of genetic and environmental factors to microbiota compositional variation, we fitted univariate ACE models to the first three principal components (PC1–3), which capture the major axes of variation in community structure (Fig. 5A). These models decompose A, C, and E, while adjusting for relevant covariates (sex, sequencing depth, batch, (and age in adults)). The proportion of variance explained by each component was estimated for infants and adults separately. In infants, shared environmental factors accounted for approximately 70% of the variance (range: [min-max]: 69–80%), with only a marginal genetic contribution (range: 0–6%). In adults, shared environmental effects were negligible, whereas variation explained by genetic factors ranged between 24–46% (Fig. 5C).

### Age-dependent genetic and environmental control of salivary microbiota predicted functions

We next aimed to assess predicted (KO) functions by quantifying within-twin-pair similarity using ICCs and decomposing variance into additive genetic (A), shared environmental (C), and unique environmental (E) components using univariate ACE twin models. ICCs and ACE models were fitted to Yeo-Johnson-transformed predicted KO function abundances represented in > 20% of the participants, adjusted for sex, sequencing depth, batch, and, in the adult analyses, age. For ACE modelling, KOs with fewer than 10 complete MZ or DZ twin pairs were excluded. Multiple testing was controlled using Benjamini-Hochberg FDR-adjusted *p*-values (Supplementary Tables 6 and 7). Overall, the KO results showed patterns similar to those observed in the species prevalence and species abundance analyses.

In infants, a total of 4024 KOs were evaluated, with overlapping ICC distributions between MZ and DZ twin pairs (*p* = 0.965) (Supplementary Fig. 2A and 2B). In adults, 5,192 KOs were assessed, and ICCs differed significantly between MZ and DZ pairs (*p* < 0.0001) (Supplementary Fig. 2C). In infants, ACE modelling indicated that shared environmental factors influenced all predicted functions (*n* = 4024), accounting for 24–77% of the variance. By contrast, host heritability contributed to variation in 53 KOs (1.3%), with estimates ranging from 20% to 43% (Supplementary Fig. 2D). In adults, the opposite pattern emerged: host heritability influenced 4589 KOs (88%) (Supplementary Fig. 2E), with A estimates ranging from 16% to 42%. Shared environmental factors influenced only four KOs, with C estimates ranging from 1% to 12%.

Among the adult KOs with the strongest additive genetic influence, many mapped to CRISPR-associated proteins and DNA/RNA processing functions, including Cas proteins and CRISPR-associated endonucleases, as well as transcriptional regulators, two-component systems, and transport or binding proteins, including multidrug efflux and small-molecule transporters. We also observed multiple KOs involved in carbohydrate and amino acid metabolism, as well as cofactor and vitamin biosynthesis.

### Heritable control of oral microbiota: caries and periodontitis taxa

The twin analyses highlighted that host genetics is a key determinant of whether adults both carry a specific bacterium and also the relative amount in the mouth. Focusing on taxa linked to the two major dental diseases (caries and periodontitis), substantial additive genetic effects (A; %, [95% CI]) on species presence were observed for many caries-associated microorganisms, including *Streptococcus mutans* (39% [24–54%])^24^ and *Bifidobacterium longum* (38% [28–48%])^25^. Likewise, carriage of multiple periodontitis-associated species showed significant genetic influence, including *Tannerella forsythia* (22% [14–31%]), *Treponema denticola* (27% [18–36%]), *Prevotella intermedia* (26% [17–34%]), and *Filifactor alocis* (32% [19–44%])^26^ and *Porphyromonas endodontalis* (33% [25–41%])^27^. Host genetics also influenced species abundance in adults, although effect sizes were generally more modest than for the presence-absence trait. In summary, these findings indicate that in adults, host genetics shapes disease-relevant oral microbiota through complementary effects on both species’ carriage and abundance (Supplementary Tables 3-6).

## Discussion

In the present study, we leveraged a large twin cohort of 3,387 twin pairs spanning infancy and adulthood to dissect genetic and environmental contributions to salivary microbiota composition, as well as single-species-level prevalence and abundance, and predicted function across distinct life-stages. Our results reveal a developmental shift, with the infant salivary microbiota and their functions predominantly shaped by shared environmental factors, whereas in adulthood, host genetics and individual-specific environmental exposures contribute substantially to microbial/functional variation. Notably, genetically influenced taxa in adults included both caries-associated and periodontitis-associated species.

Our findings that infant salivary microbiota composition is largely environmentally determined are consistent with twin-based studies reporting limited heritability and strong shared environmental effects early in life^21,28^. Cohabitation, caregiver contact, vertical transmission, and same feeding mode likely underpin the high microbial similarity observed among infant twins, in line with strain-resolved evidence of maternal-infant microbial transfer across multiple body sites^29^. In our data, shared environmental factors explained approximately 50–70% of the variation within microbial composition, with negligible genetic effects on species prevalence and only modest effects on abundance. These findings are concordant with studies in young children and mother-child pairs^16,22^.

By contrast, the divergence observed in adulthood supports a stage-dependent framework in which host genetics and individual-specific environmental exposures increasingly shape the salivary microbiota. The absence of significant shared environmental effects among adults suggests that early shared lifestyle factors contribute less to later life variability, likely reflecting the increasing influence of individual-specific exposures over the life course. As adult twins often live apart and adopt different diets, oral hygiene practices, and health behaviours, their oral environments diverge. In addition, cumulative and individual-specific factors such as antibiotic use, dental history, and local oral conditions may further shape distinct microbial communities over time. These observations may have important implications for prevention strategies: whereas early-life interventions may benefit most from environmental modification, such as diet, oral hygiene, and caregiver practices, adult oral health may require more personalized approaches that account for genetic susceptibility. Longitudinal studies, and preferentially randomized control studies, will be essential to capture temporal dynamics and disentangle causal pathways underlying these transitions.

From a clinical perspective, focusing on heritable oral taxa helps clarify the relationship between host genetics and oral disease. Previous work by Gomez et al. (2017) reported that highly heritable taxa were not necessarily associated with caries, supporting ecological models in which disease arises from environmentally driven community imbalance^30^. Conversely, Esberg et al. (2020) demonstrated substantial heritability for caries (*S. mutans*, e.g.) and several periodontitis-associated species in an independent twin setting, implicating genetic modulation of colonization or niche formation through immune or salivary traits^20^. The present findings extend this evidence by confirming notable heritability for multiple taxa traditionally implicated in dental caries and periodontal disease, including *S. mutans*, *B. longum*, *T. denticola*, *T. forsythia*, *P. intermedia*, *P. endodontalis*, *F. alocis*, and *Fusobacterium* spp. Together, these results reinforce a host genetic contribution to salivary-microbiota ecology and to disease-associated microbial consortia linked to chronic inflammation and tissue destruction^31,32^.

While environmental drivers of dysbiosis remain central, the observed heritable variance suggests that host genetic architecture may influence microbial colonization and niche formation through pathways involving innate and adaptive immunity, antimicrobial peptides, salivary glycoproteins, carbohydrate presentation, and epithelial receptor expression^19,20^. A recent genome–microbiome study^13^ supports the idea that specific human genetic variants can influence the salivary microbiota through several biologically plausible pathways. Genetic variation in *AMY1* influences starch degradation and thus the carbohydrate environment for oral bacteria, while variation in *FUT2* may alter glycan structures on oral surfaces, affecting adhesion, growth of fucose-utilizing taxa, and caries risk. Transcription factors such as PITX1 and related craniofacial regulators can change tooth morphology, enamel quality, and eruption patterns, indirectly modifying the niche available to different taxa. Immune-related loci provide a second major axis of control. *HLA* class II genes affect antigen presentation and adaptive responses to oral microbes, and TLR1 and other pattern-recognition receptors mediate innate sensing of bacterial lipoproteins and other PAMPs, shaping inflammatory pressure on colonizing species. Salivary protein genes, including *SMR3A*/*SMR3B* and the basic proline-rich proteins *PRB1*–*PRB4*, influence pellicle composition and species-specific adhesion. Together, these loci illustrate how host variation in carbohydrate processing, glycan presentation, salivary proteins, and immune recognition can selectively shape the oral microbiota.

Our observation that host genetic effects are stronger in adults than in infants is consistent with this mechanistic framework. Many of the pathways noted above, diet-related AMY1 variation, FUT2-dependent glycan landscapes, PITX-mediated dentition and enamel structure, hormone-sensitive salivary proteins such as SMR3A/B, and the full maturation of HLA- and TLR-driven immune responses, are likely to become functionally more important only after the primary and permanent dentitions have erupted, dietary complexity has increased, and the immune system and salivary glands have matured. In contrast, the infant oral environment at around one year of age is characterized by simpler tooth surfaces, a restricted diet, strong parental and environmental influence, and an immature immune system, so host genetic differences in these pathways may be less able to manifest in the microbiota. This developmental context offers a plausible explanation for why we detect robust heritable influences on the salivary microbiota in adults but not in infants.

The present study has several strengths; however, some limitations should be acknowledged. Although the large, well-characterized twin cohorts and high-resolution full-length 16S rRNA sequencing represent major strengths, genetic effects for low-prevalence taxa may be imprecise due to limited statistical power. Reliance on reference-based taxonomic assignment may under-represent novel or poorly characterized species, and participation rates may introduce selection bias. Although full-length 16S rRNA sequencing offers higher taxonomic resolution than short-read approaches, it does not capture strain-level variation. Because strains within a species can differ substantially in virulence, resistance, and fitness, species-level aggregation might obscure host genetic effects acting at the strain level. Accordingly, our heritability estimates reflect species-level signals only. Future studies using deep shotgun metagenomics with strain resolution are needed to determine whether host genetics preferentially shapes specific strains, particularly among clinically relevant taxa. Furthermore, while twin models effectively partition variance into genetic and environmental components, they do not identify the specific environmental exposures or genetic loci involved. The study was undertaken in the Swedish Twin Registry, representing a Northern European population with predominantly European ancestry. Host-microbiota interactions may be influenced by a range of factors which differ across populations, including ancestry-specific genetic variation, diet, lifestyle, and environmental exposures. Thus, the heritability estimates reported here may not be fully generalizable to individuals of non-European ancestry or to populations living in markedly different contexts. Replication in other twin cohorts with different population characteristics will be needed to assess whether these heritability estimates are stable or vary across populations. Further, the DNA isolation kit for the NewTwin and the STAGE cohorts differed, which could lead to compositional differences across cohorts. Within twin pairs, the same DNA kit was used, meaning this is not anticipated to bias heritability estimates, but limits the ability for between-cohort comparisons. The classical twin design relies on equal environmental exposure for MZ and DZ twins and on additive, independent genetic and environmental effects. Violations of these assumptions, such as unequal environments or gene–environment correlation, can inflate heritability estimates. A further limitation is the cross-sectional design, with two independent cohorts rather than longitudinal follow-up. Age-associated differences in heritability therefore cannot be associated with within-individual developmental trajectories, and cohort-specific environmental histories likely add environmental noise and make our adult heritability estimates conservative rather than artefactually large. In addition, the relatively short fasting period prior to saliva collection might mean that variability due to the preceding meal is visible in these samples. If present, this form of sampling variation is anticipated to be random with respect to genotype, which might lead to over-estimation of the non-shared environmental effect and under-estimation of the true genetic contribution. In addition, the relatively short fasting period prior to saliva collection might mean that variability due to the preceding meal is visible in these samples. If present, this form of sampling variation is anticipated to be random with respect to genotype, which might lead to over-estimation of the non-shared environmental effect and underestimation of the true genetic contribution.

In summary, our findings demonstrate that genetic and environmental influences on the salivary microbiota are strongly life-stage dependent, affecting both community structure and disease-associated taxa. Elucidating these interactions will inform precision approaches to oral health, guide the prioritization of microbial targets for prevention and treatment, and provide a framework for integrating host genetics, microbiota, and environmental exposures across the life stages. Ultimately, understanding the balance between genetic predisposition and modifiable factors in shaping the oral microbiota has broad relevance for public health, clinical practice, and mechanistic research into oral and systemic disease.

## Methods

### The Swedish twin register

The Swedish Twin Registry is the world’s largest population-based twin register^33–37^, comprising ~170,000 individuals (age 0-103) organized in nested cohorts, including the NewTwins and the STAGE cohorts used here. Basic demographic data and zygosity estimates are available for >99% of participants; overall registry participation is ~50%. Establishment of the Swedish Twin Registry was approved by the Regional Ethics Committee at Karolinska Institutet in the 1960s (https://ki.se/en/research/research-infrastructure-and-environments/core-facilities-for-research/the-swedish-twin-registry). This study was approved by the Swedish Ethical Review Authority (Dnr 2023-07572-01).

### The NewTwin cohort

The NewTwin cohort recruits twins aged 9–12 months starting in the autumn of 2021 and ongoing (https://researchdata.se/en/catalogue/dataset/2024-486). Parents are invited to participate when their twins reach 9 months of age, a time point at which they generally can recall and report on the pregnancy and birth, and where the basic health condition of the child is manifest, and, in general, they have their first teeth. After written informed parental consent, information on the infant’s early social environment, demographics, and anthropometrics, together with drooling saliva samples, are collected. The latter was primarily intended for DNA-based determination of zygosity. At the start of the present study, a total of approximately 3200 twins had been enrolled into the NewTwin, of whom 2264 had provided saliva. For this study, we used saliva-extracted DNA and demographic information.

### The STAGE cohort

STAGE is an acronym for “*Study of Twin Adults: Genes and Environment”* and represents a population-based sample of Swedish twins aged 20–47 years recruited between 2005 and 2006^35^. Of approximately 43,000 eligible twins, more than 25,000 gave informed consent and participated. Initial focus areas in the STAGE cohort included cognitive functions and dementia, cardiovascular health, metabolic disorders, mental health and well-being, and lifestyle factors such as diet, physical activity, and smoking. Data collection comprised comprehensive questionnaires on lifestyle, health, and medical history, cognitive testing, and physical examinations, alongside the collection of blood for genetic and biomarker analyses. A detailed description of the STAGE cohort is available elsewhere^35,38^. In 2009-2010, the STAGE twins were invited to donate saliva for DNA extraction. A total of 10,174 individuals provided saliva samples, and the extracted DNA was utilized in the present study.

### Saliva collection and DNA extraction

The NewTwin parents and the STAGE adults received written information on the saliva collection procedure together with a barcode-labelled collection kit (Oragene OG-500, DNA Genotek Inc., Ottawa, ON, Canada). Drooling saliva was collected by the participant or by parental assistance in infants following the manufacturer’s instructions. Briefly, eating, drinking, or chewing gum was not allowed for at least 30 minutes before sampling. Saliva was drooled into a funnel attached to a collection tube. Adults collected approximately 2 mL and for the infants for as long as the infant collaborated. Then the tube was securely capped, shaken for 5 seconds, and returned to the study center by mail. The Oragene tube contains a preservation buffer that stabilizes DNA at room temperature and is compatible with high-throughput sequencing^39^. Saliva DNA has been extracted using the Chemagen (Revvity Chemagen Technology, Baesweiler, Germany) for NewTwins and the Puregene blood kit (QIAgen, Hilden, Germany) for STAGE.

### Zygosity determination

Zygosity was determined by genotyping 46 single-nucleotide polymorphisms and evaluated as described^40^.

### Salivary microbiota characterization

The salivary microbiota was characterized from extracted DNA by full-length 16S rRNA gene sequencing according to a previously described workflow^41^. Briefly, the 16S rRNA gene was PCR amplified from position 27 to position 1492, barcoded (SQK-NBD114.96, Oxford Nanopore Technologies, Oxford, UK), pooled, and loaded onto an R10.4.1 flow cell (Oxford Nanopore Technologies) and sequenced on a GridION nanopore sequencer (Oxford Nanopore Technologies) for 72 hours. Base-calling and demultiplexing were performed on the GridION using MinKNOW (Oxford Nanopore Technologies) and the Super Accurate (SUP) model, with a quality score of > 12 and a read length between 1200 and 1800 bp. Chimeric sequences are removed during the bioinformatics preprocessing using the Porechop package, which identifies integrated adapter sequences located in the middle of the continuous read. A commercial mock community (ZymoBIOMICS Microbial Community DNA Standard, D6305, NordicBiosite, Stockholm, Sweden) with pre-purified DNA served as a positive control, and ultrapure water was used as a negative control. The reads were assigned to named (species) or unnamed but genomically defined eHOMD taxa (HMTs) (phylotype), each representing a distinct species-level 16S rRNA lineage, but for clarity, species and phylotypes are collectively referred to as species throughout the text. The EMU algorithm^42^ was used for taxonomic annotation against the eHOMD 16S rRNA database^43^. Samples with fewer than 20,000 species-matched reads were excluded, and species with <10 reads within a single sample were scored as zero to reduce false positives. For evaluating sequencing depth, rarefying to the same sequencing depth, and extracting alpha and beta diversity measurements, the R package *MicrobiotaProcess* was used. The number of species and core microbiota measurements were evaluated using the full set of participants for the NewTwin (*n* = 2264) and the STAGE cohort (*n* = 9582) (Supplementary Table 2). Alpha diversity measurements were evaluated using the Mann–Whitney U test. Differences in community composition were assessed using Bray–Curtis dissimilarity and tested by permutational multivariate analysis of variance (PERMANOVA; vegan::adonis2) with 999 permutations (group.test = TRUE). *P* values reflect differences in multivariate centroids between groups.

### Prevalence-based heritability analyses

All prevalence analyses were restricted to species present in > 5% and < 95% of samples in the NewTwin and STAGE cohorts to improve stability of variance component estimates. The prevalence-based heritability framework was first estimated using tetrachoric correlations from 2 × 2 species-presence tables, assuming an underlying bivariate normal liability, using MLE (*polycor* package), stratified by zygosity.

To decompose and estimate the variance explained by additive genetic (A), shared environmental (C), and unique environmental/measurement error (E) components, liability-threshold ACE models for binary traits were fitted in R (using the function *bptwin* from the library *mets)*, adjusting for sex, sequencing depth, and age (for adults).

### Microbial species abundance-based heritability analyses

For species abundance evaluation, taxa present in at least 5% of samples were included. For each species, abundance was Yeo–Johnson transformed (bestNormalize). Transformed values were adjusted for sequencing depth (log1p(sum_reads) and batch centred) by linear regression; residuals were used as covariate-adjusted species values for downstream ICC analyses. Single-measure intraclass correlation coefficients (ICC(1,1)) were then computed per species, stratified by zygosity, using mixed-effects models (lme4::lmer).

To disentangle genetic and environmental contributions, univariate ACE models were fitted for each species using structural equation modelling in OpenMx^44–46^. Models decomposed phenotypic variance into A, C, and E components. For each taxon, abundance was adjusted for sequencing depth and Yeo-Johnson transformed, and sex was included as a fixed effect in all models; age was additionally included for the adult cohort. Taxa represented by fewer than 10 complete twin pairs per zygosity group were excluded. Model parameters were estimated using maximum likelihood, with Hessian-based standard errors and profile likelihood confidence intervals.

### Microbiota composition: twin pair dissimilarity and determinants

To reduce sparsity, taxa present in fewer than 5% of samples were excluded. Zeros were imputed using CZM (centred log-ratio multiplicative replacement). Imputed compositions were CLR-transformed (*compositions::clr*), and to adjust for potential confounding, we residualized the CLR matrix against sex, sequencing depth, batch, and age (adults). Aitchison distance was computed in the adjusted CLR space (stats::dist(method = “euclidean”)). The *within-twin-pair* distance was defined as the unique distance between the twin pair. Results were stratified by MZ and DZ (ssDZ + osDZ). Analyses were performed in R using *dplyr*, *zCompositions*, *compositions*, *robCompositions*, and MASS. To disentangle genetic and environmental contributions to the microbiota composition, univariate ACE models were fitted for the principal components 1-3 using structural equation modelling in OpenMx as described above.

### Functional prediction and twin modelling

Functional profiles were inferred from the 16S rRNA data using the PICRUSt2 pipeline^47^. Briefly, full-length 16S rRNA gene sequence variants were used to predict functions using picrust2_pipeline.py with default settings. Predicted gene families were summarized as KEGG Orthology (KO) abundances (pred_metagenome_unstrat.tsv), and associated with KEGG pathways and higher-level functional categories via KEGG REST annotations. For each KO function, we applied a prevalence filter of 0.2, retaining only KOs present in at least 20% of samples. KO abundances were transformed using Yeo-Johnson transformation. To reduce technical variation, we residualized KO abundances for sequencing depth and batch effects before twin modelling. Sex (and age for adults) were included as a covariate in ACE modelling. For each KO, we required a minimum of 10 complete MZ-pairs and 10 DZ-pairs. Univariate ACE models were fitted in OpenMx; confidence intervals for A, C, and E were obtained from profile likelihood, when possible, with Wald approximations used as a fallback. Intraclass correlations within KO were calculated separately for MZ and DZ pairs using Pearson correlations.

### Multiple testing

To control for multiple testing, the Benjamini–Hochberg procedure was applied separately within each analysis family (i.e., prevalence and abundance ACE model comparisons)^48^. FDR-adjusted *p*-values were considered statistically significant at FDR < 0.05.

### Ethics approval and consent to participate

The study was approved by the Swedish Ethical Review Authority (Dnr 2023-07572-01) and followed the Declaration of Helsinki. All participants (STAGE) or guardians for infants (NewTwins) signed informed consent to participate in the study.

## Supplementary information

Supplementary Figures

Supplementary_Tables_1-8

## Data Availability

Research data, including salivary microbiota data, is available through the Swedish Twin Registry but is restricted by the GDPR guidelines. Please get in touch with the Swedish Twin Registry via str-research@meb.ki.se for further questions.

## Code availability

The code supporting this study is provided via the referenced R packages and other publicly available resources; additional scripts and data are available to qualified researchers upon reasonable request to the corresponding author.
