# Supplementary material for: Life stage shifts in salivary microbiota determinants: a cross-sectional study in twins
Source: NPJ Biofilms Microbiomes. 2026 Sep 16;12:174. doi: 10.1038/s41522-026-01134-0 (PMC13582848; doi:10.1038/s41522-026-01134-0)
Supplement: Supplementary file 1 [file 41522_2026_1134_MOESM1_ESM.pdf]

Supplementary Figures

Life stage shifts in salivary microbiota determinants: a cross-sectional study in twins

Supplementary Figure 1: Species richness and overall salivary microbiota composition

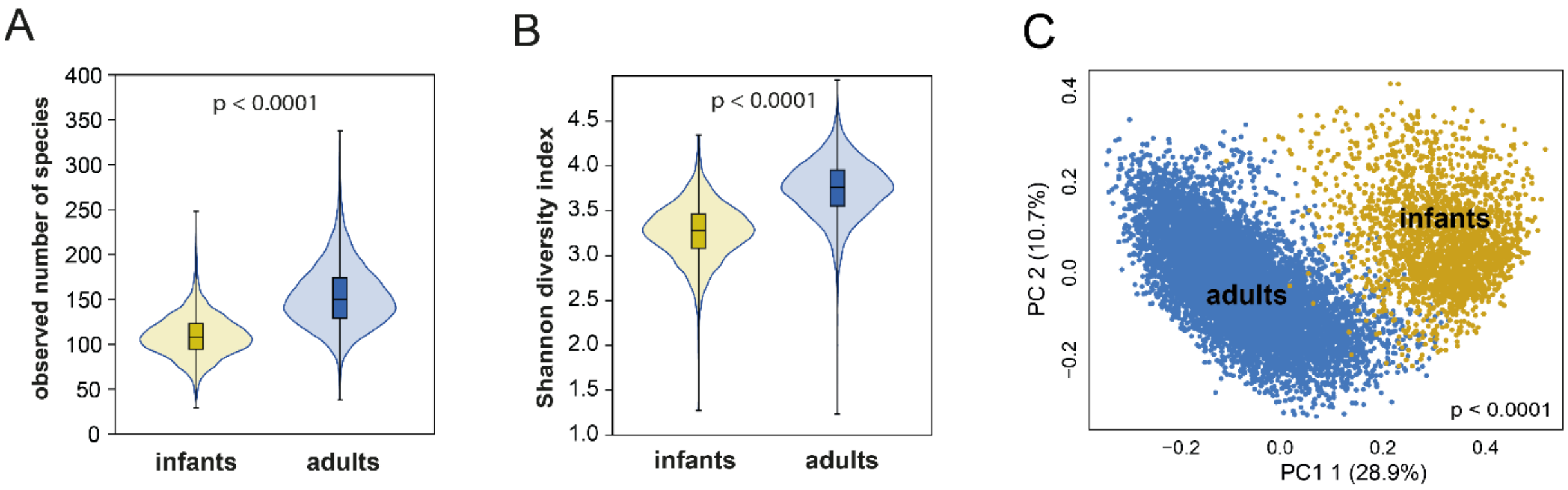

(**A**) Observed species richness. (**B**) Shannon diversity index. (**C**) Bray–Curti's dissimilarity between samples. PC = principal component; % = percent variance explained for each component. All diversity measures were calculated on data rarefied to 20,000 reads per sample. The R package *MicrobiotaProcess* was used. Alpha diversity was compared using the Mann–Whitney U test. Community composition (Bray–Curtis) was analyzed using PERMANOVA (vegan::adonis2, 999 permutations).

Supplementary Figure 2: Genetics and environmental influence on predicted salivary microbiota functions

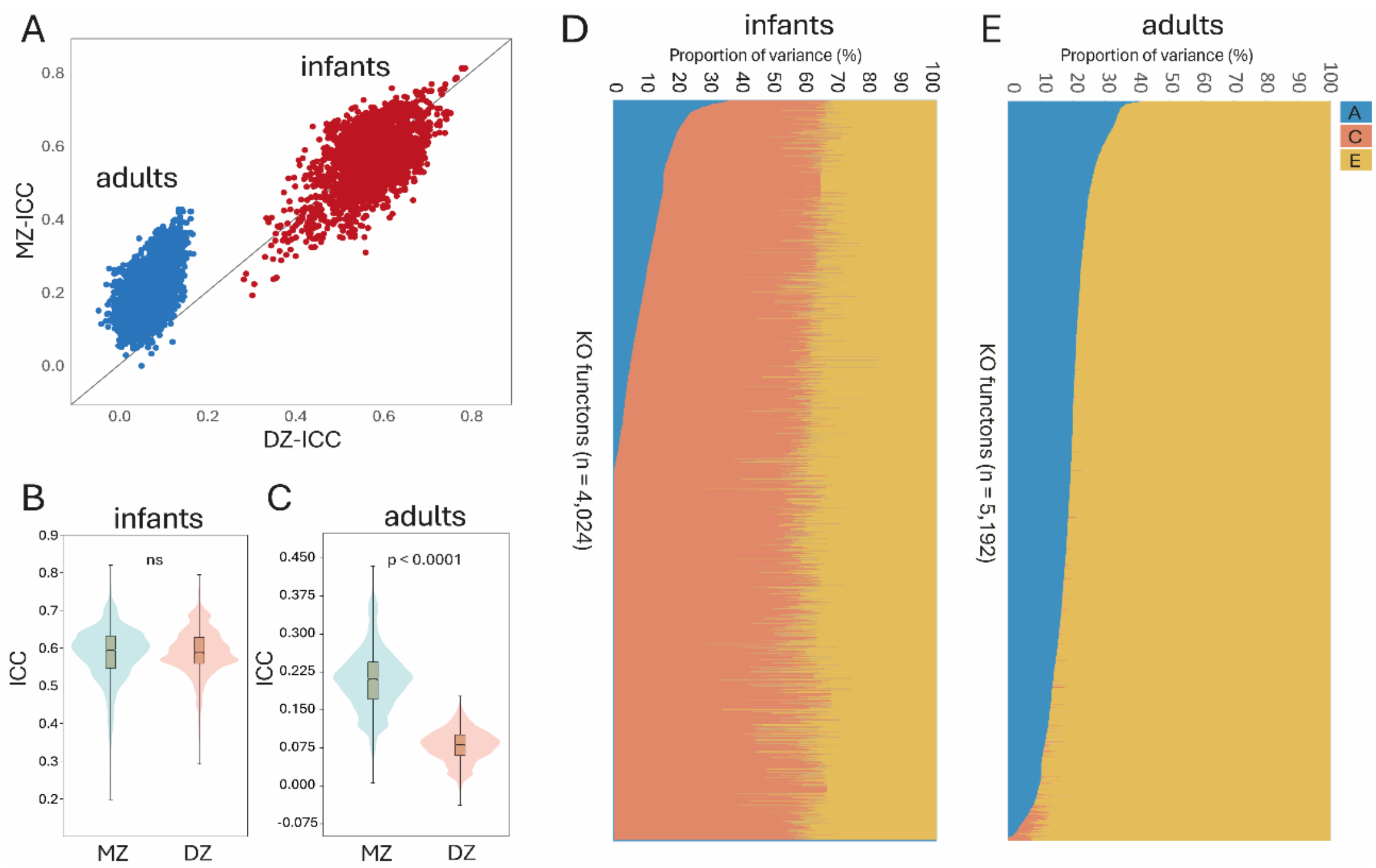

KEGG Orthology (KO) inferred from the 16S rRNA sequences using the PICRUSt2 pipeline present in >20% of participants were analysed using intra-class correlation (ICC) (**A**) in infants (**B**) and adults (**C**). Scatterplots show ICC estimates for monozygotic (MZ) and dizygotic (DZ) twin pairs; p-values indicate differences between groups (Mann–Whitney U test). Variance in KOs abundance was partitioned using ACE models into additive genetic (A), shared environmental (C), and non-shared environmental (E) components for 4,024 infant KOs (**C**) and 5,192 adult KOs (**D**). All models were adjusted for sex, sequencing depth, batch, and additionally for age in adults.
